# Peer Review of “The Performance of DeepSeek R1 and Gemini 3 in Complex Medical Scenarios: Comparative Study”

**DOI:** 10.2196/96225

**Published:** 2026-04-27

**Authors:** Jacqueline Guan-Ting You

**Affiliations:** 1 Mass General Brigham, Boston, MA, United States

**Keywords:** large reasoning model, LRM, large language model, LLM, accuracy, medical scenario, DeepSeek R1, Gemini 3

*This is a peer-review report for “The Performance of DeepSeek R1 and Gemini 3 in Complex Medical Scenarios: Comparative Study.”*

## Round 1 Review

### General Comments

This paper [1] seeks to evaluate the accuracy of DeepSeek R1 in correctly identifying the primary medical diagnosis in the medical scenarios dataset portion of Massive Multitask Language Understanding Pro (MMLU-Pro) using an open-ended format. Some clarifications on the methods and results (especially around the roles of subject matter experts vs core team members in the publication), would be helpful in understanding how these results were derived.

### Specific Comments

#### Minor Comments

1. Introduction: consider citing Deepseek AI’s Deepseek R1 paper [2].
2. Methods: please clarify who your subject matter experts were (eg, physicians, researchers) in terms of rank, specialty, and role and how they were used to grade answers (eg, selected based on specialty, 2 reviewer process, etc).
3. Methods: please indicate when the analyses were run.
4. Results: who determines whether references are related or unrelated?
5. Results and Discussion: it is unclear to me from reading the discussion portion of the paper as to whether we have any sense of whether DeepSeek R1 has correct reasoning for questions with correct diagnoses (eg, it may get the right diagnosis but may have incorrect reasoning). Similarly, did you determine the “correct answer” based on string matching (for example, if the answer was “septic arthritis” and the DeepSeek output stated “septic shock,” would this be incorrect)?
6. Discussion: consider acknowledging the sample size of questions as a limitation.

## Round 2 Review

### General Comments

The paper has been revised to address the Transparent Reporting of a Multivariable Prediction Model for Individual Prognosis or Diagnosis–Large Language Model (TRIPOD-LLM) guidelines. Overall, it appears most concerns from both reviewers have been addressed.

## Abbreviations

MMLU-Pro: Massive Multitask Language Understanding Pro
TRIPOD-LLM: Transparent Reporting of a Multivariable Prediction Model for Individual Prognosis or Diagnosis–Large Language Model
